# Philadelphia Letter

**Published:** 1880-07

**Authors:** 


### Domestic Correspondence.

### ARTICLE VIII.

### PHILADELPHIA LETTER.

*Messrs Editors* :—An event of much interest, not only to the profession of this city but of the country at large, is the recent capture—red handed, so to speak—of one of the chief manufacturers of bogus diplomas, the notorious "Dr." Buchanan. The credit of this successful effort to entrap a wary and experienced swindler is due to the enterprise of the *Record*, a penny daily paper of this city, the proprietors of which furnished the funds needed to set the machinery of law in motion. A reporter detailed by the proprietors for the purpose has spent several months in collecting the necessary evidence, and the account of his experience published with perhaps an excusable flourish of trumpets in his journal, furnishes much matter of interest.

Although the fact that Philadelphia has for some time been the center of the diploma trade is generally known, the causes which have favored this nefarious traffic, and the obstacles to its removal, are not, perhaps, so widely understood. It may not be amiss, therefore, to give some account of the rise and progress of some of these institutions. One of the oldest is in possession of a charter granted to the Pennsylvania Medical College, started some twenty-five or thirty years ago by a brilliant group of young men, most of whom subsequently became distinguished. After a brief but creditable career the institution came to an end during the late war and the faculty were scattered. What became of the charter at the time no one knows, but it was next found in the posession of some of the men who have since made a traffic of its reputation and have involved our reputable medical colleges, particularly the time-honored University of Pennsylvania, which, owing to the similarity of title has been frequently confounded with it, in unmerited disgrace, especially among foreigners. In fact as our minister to Germany remarked in his communication on the subject a few months ago, the expressive "Philadelphia degree" is even a by-word, and forms part of the furniture of a quack in popular plays. How long ago the actual sale of degrees began is not exactly known, but it became more and more a recognized business, until the "University" dwindled gradually down to little more than an office for the printing of diplomas and the transaction of correspondence connected with the traffic. After some years two sister institutions made their appearance on the scene. The "Philadelphia University of Medicine and Surgery," and the "Eclectic College." Buchanan has been the ruling spirit of the latter. Several years ago an attempt was made by the combined authorities of the University of Pennsylvania, and the Jefferson College, to bring the offenders to justice; however, not only this but subsequent efforts came to naught, the legal proceedings seeming to mysteriously fail at some point. Emboldened by impunity the diploma sellers pushed their business in foreign countries as well as in the United States, and within the last year or so several new "diploma mills" have been started. A month or two ago, however, some check was given to the traffic by the arrest of parties connected with the "Philadelphia University of Medicine and Surgery," and about the same time stimulated by the publication of Minister White's letter from Germany, the State legislature authorized the investigation of the charters of these institutions but failed to appro priate any money for the purpose of carrying on the investigation. It was about this time that the enterprising proprietor of the *Record* took the matter in hand, and in connection with the officials of the General Government, and of the State of Pennsylvania, decoyed the diploma manufacturers into the toils of the law. The reporter who undertook the work of detective, ascertained in the first place, that in addition to the three "colleges" above mentioned, no fewer than five others were at work, two of which were also situated in or near Philadelphia. From these various institutions he managed to obtain five diplomas of M.D., one degree of D.D., one of LL.D., and one of D.C.L., for the aggregate sum of two hundred and twenty-five dollars. In order to obtain these degrees various devices were employed. In one case the reporter assumed the character of a quack, printed circulars referring to his lung and liver cures, and enclosed them from Tippecanoe City, Ohio, with a letter to "Dr." Buchanan, and asking if a diploma could be procured by "writing an elaborate thesis," or by taking a three weeks' course, and expressing a willingness to "foot the bills for any risks that might be incurred." A reply from the "Dean of the Eclectic College" was soon received, and before long the correspondent was, on payment of seventy-five dollars, put in possession of no less than three diplomas. A trifling informality by which one was dated the year before the establishment of the institution granting it added to the interest. At the same time a correspondence in the name of a clergyman of Virginia was started with a view to obtaining the degree of D.D., and others. This was so successful that five diplomas, two of the M.D., one of D.C.L., one of D.D., and one of LL.D., were enclosed to a single address on payment of one hundred and forty dollars. These were captured by the United States agents, in the mails, and on the evidence thus presented, "Dr." Buchanan was arrested on the charge of using the mails for improper purposes, and was safely lodged in jail by the United States court, where he now languishes; it being apparently less easy to slip through Uncle Sam's fingers than those of our municipal and State authorities. Meanwhile, the enterprising reporter, not content with this exposure, was carrying on several other schemes for entrapping the diploma mongers, one of which is most amusing as recited by the chief actor.

It appears that among the more recently fledged institutions of learning which have been created in this city is one known as the "Philadelphia Electropathic Institution." The building occupied by this learned body is a plain, three-story house, with a small sign on the front door, which your correspondent has read on passing many times in the last fifteen years without suspecting the latent possibilities concealed behind so unpretentious an exterior. In fact, for many years there was nothing more to be seen inside than an ordinary waiting room and office filled with batteries. But the recent successes of Buchanan had incited the proprietors of the establishment to follow in his footsteps, and advertisements appeared in the papers offering instruction and diplomas. Under the guise of a countryman the reporter applied for instruction and attended the " full course " of the institution, comprising seven lectures, each about an hour in length, and each reiterating what had previously been said on the " Theory of Electricity." At the end of a week the "professor " said he was going out of town and that the course of lectures would now be closed. The student was provided with a work upon medical electricity and a copy of Cutter's Anatomy. One week's reading with a lecture on the "winker" muscles of the eye concluded the course, and having prepared a thesis, the student was examined, the questions being as follows: "How would you diagnose?" "How would you treat a headache?" "How would you treat a case of eruption?" "How would you treat neuralgia?" "What would you say if some one came in your office and said, 'electricity is nothing?' " The reporter's answers to these questions were so satisfactory that the professor assured him with effusion that he "had grasped the philosophy and treatment better than any student they had had," and, on payment of one hundred and thirty dollars the "right hand of fellowship" was proffered the newly fledged graduate and an elaborate diploma (given in the *Record* in fac simile), was presented him, declaring him, in sonorous latin phraseology, "In Electro-Therapeuticis Magistrem." It is estimated that the profits of this institution from the sale of diplomas must range from three to four thousand dollars a year. The law seems powerless to prevent the establishment of these institutions; all that could be done in this case when the charter was applied for a few months ago, was to reduce the power of giving degrees, so that only that of master in electricity might be granted instead of doctor, as was desired. Three or four similar institutions have applied for the power of giving degrees during the past few weeks, and had not a check been given to their proceedings by this exposure, it is likely we should have been overrun by a small host of " diploma mills" before the close of the year. The fact is that the abortive attempts to expose and punish the diploma vendors have simply served to advertise them as sources and this city as a center at which such degrees could be obtained. The profession of this city has been blamed for not taking more active steps to ferret out and destroy this nefarious traffic, but in extenuation it must be said that previous attempts having failed through what appeared to be the connivance of the municipal legal authorities, no new efforts could be made so long as the fountain of justice itself seemed to be poisoned and the officers of the courts, to all appearances, in league with the guilty parties. An incident may be mentioned in illustration of the impudent boldness with which these bogus diplomas were flourished under the noses of the public. In is said that when Buchanan was a candidate for same petty office in his ward, medical diplomas were freely offered among the colored population with the view of influencing that vote.

In capturing the "faculty" of the Electic Medical College recourse was had to the United States authorities as being more certain than those of the municipality or State, although some of the latter assisted. The charge upon which Buchanan was arrested was that of using the United States mails for purposes of fraud, and when the officers of the law, armed with the evidence afforded by the exterprising reporter of the *Record*, entered the "college" the "dean" was discovered surrounded by piles of signed and unsigned diplomas, the seals of the various learned institutions under his charge and a mass of correspondence bearing on the diploma traffic. Thus far no responsible bail has been offered, and Buchanan is still in jail, but what will be the final result of the case is very difficult to say. The charge under which he has been committed does not touch the character of his institution, and it is very much to be feared lest, when the penalty of this misdemeanor has been paid, the business will once more be resumed, unless advantage shall be taken of the opportunity now afforded to have the charter revoked. Among the "faculty" of the "Electic Medical College" is one name, that of Charles G. Polk, which some of your readers may remember in connection with "glycerite of kephaline," a mess concocted by this worthy and lauded by him in all the journals so long as he could obtain admission to their columns. The true character of the compound and its author were well shown up by Dr. Samuel R. Percy, of New York, until he became generally known, when he left the ranks of the regular profession and turned quack.

We have had two medical (or surgical) sensations since my previous letter, the audiphone and bromide of ethyl. The former of these I may have mentioned. It was much talked about for a time, and some of the daily journals contained the papers read before the County Medical Society, a species of advertisement more glaring than any heretofore practiced in this locality. Otologists, I believe, united in speaking lightly of the advantages of the audiphone, but possibly the objects of its promotors were attained in the temporary notoriety gained by its general advertisement in connection with their names. At any rate it was soon dropped. Bromide of ethyl has also, it seems, had its day. Brought forward, in part, by the persons by whom the audiphone had been *exploited*, it has already been the cause of one death in the hands of a prominent surgeon of the city, and seems likely to become very unpopular. PHILA.

---

IF pepsin is dissolved or suspended in any fluid of neutral reaction its power of digestion is interfered with; that means, it cannot show its full strength. If, however, the solution is made alkaline, the pepsin at once becomes inert—it loses all power to change albumen into pepton. The proper relation between some kind of acid—hydrochloric or lactic to be preferred—and the pepsin is an essential condition to bring out its digestive power, and, in therapeutics, the good effects of pepsin. Not only as an exception, but we well might say as a rule, we find in looking over prescriptions which contain more or less of pepsin, that the practitioner tries his best to combine pepsin with a variety of vegetable or mineral substances in such a manner that it cannot show its digestive power at all. Nothing is more common than to see pepsin combined with snbcarbonate or subnitrate of bismuth, and yet, it can easily be shown that the addition of even the latter salt to artificial gastric juice will interfere with the digestion of egg albumen. Bicarbonate of soda, the different preparations of iron, strong alcoholic tinctures, and elixirs are incompatible with pepsin.—*Kretzschmar.*

